# Authors’ Response to Peer Review of “Using Electrooculography and Electrodermal Activity During a Cold Pressor Test to Identify Physiological Biomarkers of State Anxiety: Feature-Based Algorithm Development and Validation Study”

**DOI:** 10.2196/77440

**Published:** 2025-07-10

**Authors:** Jadelynn Dao, Ruixiao Liu, Sarah Solomon, Samuel Aaron Solomon

**Affiliations:** 1Computer Science, California Institute of Technology, Pasadena, CA, United States; 2Medical Engineering, California Institute of Technology, 1200 E California Blvd, Pasadena, CA, 91106, United States, 1 626 395 6811; 3Adult Psychiatry, Dartmouth College, Hanover, NH, United States

**Keywords:** stress, biomarker discovery, EOG, EEG, medical informatics, electrooculography, electroencephalogram


*This is the authors’ response to the peer-review report of “Using Electrooculography and Electrodermal Activity During a Cold Pressor Test to Identify Physiological Biomarkers of State Anxiety: Feature-Based Algorithm Development and Validation Study.”*


## List of Major Concerns and Feedback

### Concerns With Methods


*It would be helpful to document the name of the device and manufacturer used in this study to record the electrooculography (EOG). This would help other researchers who may want to reproduce the results.*


**Response:** We appreciate the reviewer’s [1] suggestion and agree that providing this information would improve the reproducibility and clarity of our study [2]. We have now added the name of the EOG device and its manufacturer in the Methods section of the revised manuscript. The updated text reads as follows:

“Our setup integrated the AD8232 (Analog Devices), a biopotential amplifier designed to capture physiological signals, which we optimized for measuring EOG activity.““Additionally, 19 trials lasting between 30 seconds and 2 minutes were conducted under conditions with no blinking, but with deliberate wire movements introduced by manually adjusting or lightly tugging the electrode leads.”“EOG recording used the same setup as the Blink Identification EOG Dataset (BLINKEO) data collection. Electrodes were positioned above and below one eye to detect vertical eye movements by capturing corneo-retinal potential shifts.”


*Similarly, it would be helpful to add additional details about the cold pressor test (CPT) methods. For example, was a commercially available circulating water bath used to maintain a constant water temperature? Was the temperature of the subject’s hand monitored? The details of the cold stressor test (the water temperature, the period of immersion, and the cutoff point) should be added for the sake of clarity, transparency, and reproducibility. Past studies using these metrics should also be referenced for details (eg, [3]). These methodological details may also be added in the form of a figure to add clarity to the experimental setup.*


**Response:** In response, we have expanded the Methods section to include additional details about the CPT setup. First, the reference that was suggested in the reviewer’s comment was added. In response, we have also expanded the Methods section to provide a clearer description of the CPT protocol. Specifically, we now included “In the cold-water trials, participants immersed their hand in a circulating water bath set to a constant temperature of 0-6°C. Participants maintained immersion for approximately 5 minutes or until voluntary withdrawal.” Furthermore, we have removed mention of exercise trials, as they were not used in dataset creation or analysis and are thus not relevant to the study.


*To better understand the individual response to the cold challenge before participating in the actual experiment, it is advised that the manuscript states what type of participant testing was or was not adopted in the cold pressor testing experiment. For example, what were the tolerance times? Were there any gender differences? If any pretesting data were collected, analyzing them and presenting them as results would add clarity to the results.*


**Response:** We did not implement a formal pretesting phase to assess individual tolerance times before the experiment. All participants were instructed to immerse their hand in the CPT until they reached their tolerance limit or approximately 5 minutes (300 seconds). A summary of trial durations for each phase of the experiment—baseline (before hand submersion), CPT (cold water immersion), and recovery (after hand removal)—is presented in Table 2c. This table includes the minimum, 25th percentile, median, 75th percentile, and maximum tolerance times recorded across participants. Table 2c’s description was amended to make this more clear:

“d. Summary of the duration of time EDA and EOG features are collected from, across different experimental phases. For each phase—Baseline (before hand submersion), Cold Pressor Test (cold water immersion), and Recovery (after hand removal) —both tables list the minimum, 25th percentile, median, 75th percentile, and maximum duration (in seconds).”

Regarding gender differences, our study was not explicitly designed to analyze gender-based variations in cold stress tolerance, and the sample size for gender-based comparisons is limited. However, we acknowledge the potential relevance of such analyses and have noted this as an area for future investigation in the Conclusion:

“An important next step is to investigate potential gender-based and race-based differences in physiological responses to acute stress and our current methods of inducing stress, as our current study was not explicitly designed for such analysis but acknowledges its relevance.”


*It is unclear if the 65 repeating blinking trials and the 19 no-blinking trials were collected from the same individual or from different individuals. Please clarify.*


**Response:** We agree that clarifying whether the trials were conducted on the same or different individuals improves the transparency of our methodology. In the revised manuscript, we have explicitly stated that all trials were conducted on the same individual to ensure consistency in signal characteristics. The updated text now reads “All trials were conducted on the same two individuals for consistency in signal characteristics.”


*No signal voltage/electrical records for electrodermal activity [EDA] were found in the manuscript. Is this intentional? Please consider adding this information.*


**Response:** In the revised manuscript, we have now explicitly provided details on the EDA signal acquisition, including the applied voltage and electrical characteristics. The updated text reads as follows:

“EDA signals were recorded using a GSR (Galvanic Skin Response) sensor with MCP606 (Microchip Technology) operational amplifiers, operating at an excitation voltage of 0.5V to measure skin conductance. Electrodes were placed on the forehead, chosen for its sensitivity to stress-induced sweat gland activity. The recorded signals were digitized and processed in real-time using an ESP32-S3 WROOM-1 (Espressif Systems) microcontroller, which managed data acquisition, signal processing, and wireless transmission.”


*It would be important to add details of ordinal variables present in the Positive and Negative Affect Schedule (PANAS) and the State-Trait Anxiety Inventory (STAI-State), and clearly state their function and use in Supplementary Table 2.*


**Response:** In response, we have updated Supplementary 2’s table to explicitly describe how these scales function in the assessment of emotional and anxiety states. The revised descriptive text in Supplementary 2 now reads:

“The survey items from the Positive and Negative Affect Schedule (PANAS) and the State-Trait Anxiety Inventory (STAI-State) were used to assess participants' emotional and anxiety responses during the experiment. The PANAS scale consists of 10 items measuring Positive Affectivity and Negative Affectivity, each rated on a 1-5 Likert scale, where higher scores indicate stronger affective states. The STAI-State consists of 20 items assessing state anxiety, measured on a 1-4 Likert scale, where responses indicate varying degrees of agreement with statements reflecting anxiety levels. Higher scores in negative affectivity and anxiety-related items indicate greater distress, while higher scores in positive affectivity items indicate greater emotional well-being.”

### Concerns With Analysis


*F_1_-scores that were mentioned in the text (87.34% and 79.99%) are not present within the figures. Moreover, an F_1_-score is an integer value from 0 to 1, taking precision and recall into account, and is not often expressed as a percentage.*


**Response:** The updated text now expresses the *F*_1_-scores as decimal values, aligning with the conventional representation. In addition, the figures now include the accuracy and *F*_1_-score: “0.8734” and “0.7999.”


*Figure 1c has two separate graphs; it should be captioned as 1c and 1d. What do both these graphs portray? The second graph for 1c is missing titles for the x- and y-axes—the current assumption is that they are the same as the first graph.*


**Response:** The figure has been updated to distinctly label the two separate graphs as Figure 1c and Figure 1d in both the figure and the caption. We clarified the purpose of both graphs, stating that they each depict independent blink events, highlighting the variability in peak shape that can occur in EOG recordings:

“d. Another example of a blink peak, demonstrating the variability in blink peak shapes observed across recordings. The feature extraction process remains consistent, with boundaries determined by identifying the nearest minima on either side of the peak.”


*Table 1 lacks a legend and is shown as panel a of Table 2. Please check how the tables are referenced in the text to make sure they reference the right one.*


**Response:** Table 1 is now correctly referenced in the manuscript to ensure clarity. A brief description has been included to clarify its contents, explicitly stating that it summarizes the trial characteristics, total duration, and peak detection results before and after filtering.

“Table 1 summarizes the characteristics of these trials, including session count, total recording time, and peak detection results before and after filtering.”

We have verified all text references to ensure that Table 1 and Table 2 are cited appropriately.

“Sixteen participants (N=16) between ages 26-31 took part in the study, and demographic information, including race and gender, was collected and is summarized in Table 2a-b. Each trial lasted about 10-15 minutes and was divided into three phases: baseline, CPT (Cold Pressor Test), and recovery. The length of the trial and the data used for feature analysis is as detailed in Table 2c-d.”


*The captions of the figures should have statistical information when relevant. For example, in Figure 3, the caption should include a description of what data were plotted and the meaning of the graph. Presumably plotting medians, quartiles, and SDs? Also, please report n values.*


**Response:** Figure 3 has been updated to include the median and SD of each score. Figure 2 has been updated to include accuracy and *F*_1_-score for each culling step.

### Concerns With Ethics


*It is not clear what the ethical statement at the end of the manuscript, which states that the study was exempt from review board approval, means. That statement should be revised for clarification. In addition, details regarding whether or not institutional review board approval was obtained, whether the study involved consenting participants and used humans, how the data were collected and used, how the data were handled to protect the privacy of study participants, and any other ethical procedures that were followed to protect subjects from any harm due to participation in the study should be added.*


**Response:** We have clarified the ethical statement at the end of the manuscript. This study was conducted in accordance with ethical guidelines for research involving human participants. All patient data were fully anonymized prior to analysis, with identifying information removed and data transmission secured using byte-splicing encryption methods. All participants provided informed consent for the use of their data in this study. The study adhered to data privacy and security protocols to ensure the confidentiality and protection of participants.

## List of Minor Concerns and Feedback

### Minor Concerns With Methods


*Please document whether the data were taken from each subject only once or whether data were obtained several times from a subject.*


**Response:** In response, we have explicitly stated that data were collected from each subject only once in the revised manuscript. The updated text now reads:

“Sixteen participants (N=16) between ages 26-31 took part in the study, and demographic information, including race and gender, was collected and is summarized in Table 2a-b. Data was taken from each subject only once.”


*Referring to the line “To focus on blink-like events, we applied criteria based on established blink characteristics,” the criteria used to establish blink characteristics should be cited, if not already given.*


**Response:** To address this, we have now clarified how we derived this criteria. The revised text now references the methodology of BLINKER, a pipeline for extracting ocular indices such as blink rate, blink duration, and blink velocity-amplitude ratios from electroencephalogram channels, EOG channels, and/or independent components.


*Shapley additive explanations (SHAP) analysis was performed on combinations of 5 features. Please clarify on what basis these 5 features were chosen (out of 15 of EDG and 33 of EOG).*


**Response:** We have clarified the description of the SHAP analysis methodology:

“In this study, SHAP analysis was performed on combinations of five features, selected from the total feature set of 15 EDG and 33 EOG features, highlighting the significance of how certain biomarkers, used together, reveal more prominent interactions and effects on model predictions. This approach underscores that certain biomarkers, while potentially less impactful individually, can demonstrate substantial importance when analyzed as part of a group. By evaluating these interactions, we understand how combinations of features can provide insights into the model’s behavior that single-feature analyses might overlook.”“The quality of a set of features is determined by considering their collective contribution to the model’s predictions, measured through the mean absolute SHAP values across the dataset. A high-quality set of features is one where the combination of features demonstrates substantial importance, as indicated by a higher mean absolute SHAP values. This benchmark reflects not only the magnitude of individual contributions but also the degree to which the features, as a group, interact to enhance the predictive power of the model.”

### Minor Concerns With Analysis and Presentation


*Page 10, Electrooculography (EOG) Signal Segmentation section: the authors mentioned that they extracted 33 features; however, Supplementary 4 mentioned 35 feature definitions. Please revise and correct.*


**Response:** We have cross-checked the manuscript and Supplementary 4 to ensure consistency in the reported number of features. A total of 35 features were used, so we have revised the EOG Signal Segmentation section to correctly state “35 features” instead of “33.”


*In Figure 3, please put “STAI-State survey score” on the y-axis for clarification rather than just “Scores.” In addition to box and whiskers plots, adding column graphs for positive affectivity, negative affectivity, and s-anxiety might be beneficial to more clearly express the SD present within the data.*


**Response:** We agree that column graphs can effectively complement the box plots by visually emphasizing SDs within the dataset. We have introduced bar charts with error bars to represent mean survey scores for each stage (baseline, CPT, and recovery). The axes and labels were also clarified, per request. The figure description now includes:

“Figure 3 User-reported survey responses during each stage of the trial, displaying both box-and-whisker plots and column graphs for Positive Affectivity, Negative Affectivity, and State Anxiety (S-Anxiety) across the Baseline, CPT, and Recovery stages.”


*It would be beneficial to graphically display the F_1_-scores that were collected across the study.*


**Response:** We have updated Figure 2 to include the *F*_1_-scores across each step of the culling pipeline.


*The figures are quite small, which makes readability a little difficult. Please make the text larger to improve readability and accessibility.*


**Response:** Figure axes labels, headings, and some descriptions were adjusted with larger text.


*The Figure 1a description states, “The red dotted lines indicate the center of the peak…,” but these appear to be gray.*


**Response:** We have resolved this figure description, which now reads, “The grey dotted lines…”

### Suggestions


*Consider the inclusion of a Limitations section in this manuscript to better discuss potential limitations due to the skewness in male and female participants, data curation, applied methodologies, and other limitations of the study.*


**Response:** A “Limitations” section was added to this manuscript in the Conclusion. It reads “This study advances state anxiety biomarker detection using Electrooculography (EOG) and Electrodermal Activity (EDA), but several limitations should be noted. The participant pool (N=16) was demographically skewed, with a predominance of male and Asian participants, limiting generalizability. Data was collected only once per subject, preventing analysis of intra-individual variability over time. Future studies should incorporate larger and more diverse populations with longitudinal data.

“The Cold Pressor Test (CPT) was conducted in a controlled lab environment, which may not fully reflect real-world anxiety triggers. Additionally, motion artifacts in EOG recordings, despite filtering efforts, could impact signal clarity. EDA signals were recorded using a single forehead electrode, though different placements (e.g., fingertips) may improve accuracy. Improved artifact detection and additional motion-tracking sensors could enhance data quality. Feature selection for SHAP analysis focused on optimizing interpretability, but alternative selections may yield different insights. Models and analyses constructed using this dataset may not generalize well to other stress-inducing scenarios. External validation using independent datasets is necessary to confirm these findings.”


*A figure showing the trial structure would be very useful to understand how the data were collected.*


**Response:** The design of these trials facilitated the collection of time series data during an environmental stressor. We have added an additional figure to make the setup/timeline of this experiment more clear:

“Figure 2 This figure presents a visual representation of the experiment timeline, detailing the Baseline, Cold Pressor Test (CPT), and Recovery phases. The raw Electrooculography (EOG) and Electrodermal Activity (EDA) signals across these phases show no immediately clear trend distinguishing the baseline and recovery from the CPT stressor. However, when specific features such as Blink Duration from EOG and Hjorth Activity from EDA are extracted and overlaid, more distinct patterns emerge, and can be used to quantify physiological responses to stress induction and subsequent recovery.”

## References


*In the third paragraph of the Introduction, adding a reference to other techniques used to provoke anxiety, including the reduced EDA response in depressed patients, and the conflicting studies could be helpful to the readers.*


**Response:** Four additional references were made to cite techniques that have been shown to provoke anxiety. Also, additional sentences were added to discuss the response variability introduced by depression, medication usage, and methodological differences:

“Electrodermal activity (EDA) is a common measure of physiological arousal, but its reliability in depression research remains debated. Some studies report reduced EDA responses in individuals with major depressive disorder, suggesting impaired autonomic reactivity12 and emotional hypo-responsiveness13. However, conflicting findings point to variability due to factors like medication use and methodological differences14, emphasizing the need for further research on the relationship between physiological signals and emotional states.”


*In the Introduction, fourth paragraph, the reference “Schachter and Singer” is not present in the References. Is this the wrong reference, or it just needs to be added to the list?*


**Response:** Schachter and Singer [4] has now been added to the list of references.


*In the Introduction, third page, third paragraph, it is advised to add references to document the reduced EDA response in depressed patients and the conflicting studies.*


**Response:** This comment is a repeat of the first comment in the References section and was addressed accordingly.


*In the Methods, please cite sources for the Butterworth filter (page 5), the Savitzky-Golay filter (page 5), and all other analyses.*


**Response:** Specifically, we now reference Virtanen et al [5] for the implementation of these filters in the SciPy library. Additional citations have been included where applicable to provide proper attribution for the analytical techniques used.


*Reference 2: Include full citation with a link.*


**Response:** This was corrected.


*Reference 3: It is advised to correct the article name to “APA 2023 Stress in America Topline Data.”*


**Response:** This was corrected.


*Reference 4: The correct citation should be “Kazanskiy NL., Khonina S.N., Butt M.A. A review on flexible wearables—Recent developments in non-invasive continuous health monitoring. Sens. Actuators A Phys. 2024;366:114993. doi: 10.1016/j.sna.2023.114993.”*


**Response:** This was corrected.


*Reference 10: The correct citation should be: “Electrooculogram Analysis and Development of a System for Defining Stages of Drowsiness Master's Thesis Project in Biomedical Engineering, Linköping University, Dept. Biomedical Engineering, LiU-IMT-EX-351 Linköping 2003. Available: https://www.diva.portal.org/smash/get/diva2:673960/FULLTEXT01.pdfTest.”*


**Response:** This is now reference 16. This was corrected.


*Reference 19: The correct citation should be “Anxiety Detection Using Multimodal Physiological Sensing, 2021 IEEE EMBS International Conference on Biomedical and Health Informatics (BHI), Athens, Greece, 2021, pp. 1-4, doi: 10.1109/BHI50953.2021.9508589.”*


**Response:** This is now reference 25. This was corrected.


*Reference 23: Revising this citation is advised as searching on the internet shows error 404. The requested URL was not found on this server. Moreover, this is not a proper citation—give the edition number of the book (there are at least 5 editions) and publication year, as well as the page number of the cited data point about typical blink elapsed time.*


**Response:** This is now reference 29. This was corrected.


*Reference 27: The correct citation should be “Hassanein, A.M.D.E., Mohamed, A.G.M.A. & Abdullah, M.A.H.M. Classifying blinking and winking EOG signals using statistical analysis and LSTM algorithm. Journal of Electrical Systems and Inf Technol 10, 44 (2023). https://doi.org/10.1186/s43067-023-00112-2”*


## References

[1] Saderi D, Rasania S, Olatoye T (2025). Peer review of “State Anxiety Biomarker Discovery: Electrooculography and Electrodermal Activity in Stress Monitoring (Preprint)”. JMIRx Med.

[2] Dao J, Liu R, Solomon S, Solomon SA (2025). Using Electrooculography and Electrodermal Activity During a Cold Pressor Test to Identify Physiological Biomarkers of State Anxiety: Feature-Based Algorithm Development and Validation Study. JMIRx Med.

[3] Mitchell LA, MacDonald RAR, Brodie EE (2004). Temperature and the cold pressor test. J Pain.

[4] Schachter S, Singer JE (1962). Cognitive, social, and physiological determinants of emotional state. Psychol Rev.

[5] Virtanen P, Gommers R, Oliphant TE (2020). SciPy 1.0: fundamental algorithms for scientific computing in Python. Nat Methods.

